# Confidence-Based, Collaborative, Distributed Continual Learning Framework for Non-Intrusive Load Monitoring in Smart Grids

**DOI:** 10.3390/s25123667

**Published:** 2025-06-11

**Authors:** Chaofan Lan, Qingquan Luo, Tao Yu, Minhang Liang, Zhenning Pan

**Affiliations:** The School of Electrical Power Engineering, South China University of Technology, Guangzhou 510640, China; eplanchaofan@mail.scut.edu.cn (C.L.); epqqluo25@mail.scut.edu.cn (Q.L.); epmhliang2024@mail.scut.edu.cn (M.L.); scutpanzn@163.com (Z.P.)

**Keywords:** Non-Intrusive Load Monitoring (NILM), distributed continual learning, catastrophic forgetting, confidence-based collaboration, lightweight architecture

## Abstract

Non-Intrusive Load Monitoring (NILM), a technique that extracts appliance-level energy consumption information through analysis of aggregated electrical measurements, has become essential for smart grids and energy management applications. Given the increasing diversification of electrical appliances, real-time NILM systems require continuous integration of knowledge from new client-side appliance data to maintain monitoring effectiveness. However, current methods face challenges with inter-client knowledge conflicts and catastrophic forgetting in distributed multi-client continual learning scenarios. This study addresses these challenges by proposing a confidence-based collaborative distributed continual learning framework for NILM. A lightweight layer-wise dual-supervised autoencoder (LWDSAE) model is initially designed for smart meter deployment, supporting both load identification and confidence-based collaboration tasks. Clients with learning capabilities generate new models through one-time fine-tuning, facilitating collaboration among client models and enhancing individual client load identification performance via a confidence judgment method based on signal reconstruction deviations. Furthermore, an anomaly sample detection-driven model portfolios update method is developed to assist each client in maintaining optimal local performance under model quantity constraints. Comprehensive evaluations on two public datasets and real-world applications demonstrate that the framework achieves sustained performance improvements in distributed continual learning scenarios, consistently outperforming state-of-the-art methods.

## 1. Introduction

The increasing global energy demand and advancement of “dual-carbon” goals have made refined power system management and efficient energy utilization critical priorities. Non-Intrusive Load Monitoring (NILM), an innovative power monitoring technique proposed by Hart in 1980 [1], enables non-invasive identification of appliance states in residential or industrial environments through analysis of aggregated electrical signals from the main meter. Compared to traditional intrusive methods requiring individual device sensors, NILM provides significant advantages including lower deployment costs, enhanced client acceptance, and simplified maintenance, establishing it as a fundamental technology for smart grids and energy management [2,3].

NILM algorithms can be broadly categorized into event-based and event-free approaches [4]. Event-based methods detect power transients triggered by appliance state transitions, extract corresponding waveform features, and determine load types through load identification models [5,6]. Event-free methods focus on modeling steady-state characteristics or utilize deep learning for load disaggregation [7,8]. This research emphasizes event-based NILM due to its: (1) enhanced adaptability to diverse appliances, aligning with increasing appliance heterogeneity; (2) strong interpretability, as detected electrical features directly correspond to appliance behavior; (3) real-time capability, making it appropriate for dynamic scenarios such as industrial equipment switching.

Current NILM research predominantly focuses on the pre-deployment training phase, where models are developed using comprehensive load datasets from controlled environments such as residential settings [9,10]. However, post-deployment, two significant distributional shifts emerge: (1) introduction of new appliances that were unseen during the training phase, and (2) cross-scenario generalization challenges where models trained in one domain (e.g., residential settings) are applied to another (e.g., industrial loads) with markedly different load characteristics [11]. These disparities between training and real-world data distributions inevitably compromise model performance [12]. Conventional approaches including transfer learning [13] and meta-learning [14] propose optimizing models using labeled data from client-side appliances or new scenarios. A typical implementation involves pre-training feature extraction layers followed by fine-tuning the fully connected layers with client-side data [15,16]. Advanced studies further achieve unsupervised domain adaptation through source target domain data distribution alignment [17]. The principal advantage of these methods lies in their ability to reduce dependence on target clients’ training samples through pre-trained models. However, they exhibit two critical limitations in practice: (1) The assumption of static client appliance datasets permits only single-round parameter updates without accounting for continuous data distribution shifts. (2) The presumption that all clients can provide labeled new data for learning. Additionally, privacy protection regulations restrict sharing or uploading locally labeled data to centralized servers, confining such data to local use [18,19]. These limitations pose a novel challenge: acquiring new data knowledge from clients with labeled data and learning capabilities while enhancing the load identification performance of the entire system, particularly for non-adaptive clients lacking learning conditions.

To address the aforementioned challenges, a fundamental solution involves adopting standard continual learning methods [20]. This approach treats each learnable user as a distinct learning task within the continual learning paradigm, where models transfer between learnable users while executing continual learning algorithms. This method suffers from potential alteration of established mapping relationships during each learning phase, leading to catastrophic forgetting of previously acquired user knowledge [21] and hindering knowledge accumulation. Researchers have proposed constraining updates to critical parameters to protect knowledge obtained from prior users [22,23]. Nevertheless, cumulative parameter modifications during extended learning processes still risk gradual knowledge erosion. Alternative strategies include knowledge distillation integration [24], where teacher models are preserved after each learning phase and consistency losses are incorporated during subsequent training to maintain output alignment with teacher models. Another common approach involves retaining partial historical samples for participation in new learning tasks [25]. However, sample preservation necessitates data transmission between users, potentially violating privacy preservation principles. Consequently, standard continual learning methods face either privacy compliance limitations or forgetting issues in distributed scenarios. Considering user distribution characteristics, an associated alternative involves federated learning integration [26]. For this method, each learning iteration involves simultaneous participation from both new and historical clients, with global model updates achieved through parameter aggregation. Although this method preserves prior knowledge, it incurs computational and communication costs that increase linearly with the growing number of participating clients per iteration [27]. In summary, both sequential learning and federated learning demonstrate limitations in achieving sustained knowledge accumulation within distributed and incrementally expanding data environments. A key constraint stems from their dependence on single-model architectures, which require ongoing parameter adjustments during extended learning periods, inevitably compromising early-stage knowledge. 

This paper introduces a confidence-based, collaborative, distributed continual learning (CBCDCL) framework to address existing challenges in non-intrusive load monitoring. The framework represents the first distributed continual learning approach for NILM that effectively manages knowledge conflicts and forgetting issues in distributed client-specific learning environments, while facilitating cross-client collaboration to improve load identification performance across the system—particularly benefiting clients without learning capabilities. 

This study proposes a confidence-based, collaborative, continual learning framework for Non-Intrusive Load Monitoring (NILM), which enables clients with local learning capabilities to rapidly generate sub-models through one-time fine-tuning. The framework subsequently achieves continuous improvement of system monitoring performance through model collaboration. Three core innovations are integrated: (1) a lightweight, layer-wise, dual-supervised autoencoder that combines a fully connected architecture with layer-wise dual-supervision training, achieving efficient electrical load feature extraction for both identification and confidence judgment; (2) an anomaly sample detection-driven dynamic model portfolio update method, which customizes and continuously optimizes local model portfolios under computational constraints; (3) a confidence-based collaborative mechanism that leverages signal reconstruction deviation to enable cross-client knowledge transfer. Experimental validation on two public datasets and real-world deployments with STM32F407-based smart sockets demonstrates the framework’s capability to incrementally accumulate distributed learnable data while balancing computational overhead and improving identification accuracy. These contributions collectively address the challenges of adaptability and sustainability in event-based NILM systems under distributed continual learning scenarios.

## 2. Problem Statement

### 2.1. Event-Based NILM Methodology

**Figure 1:**
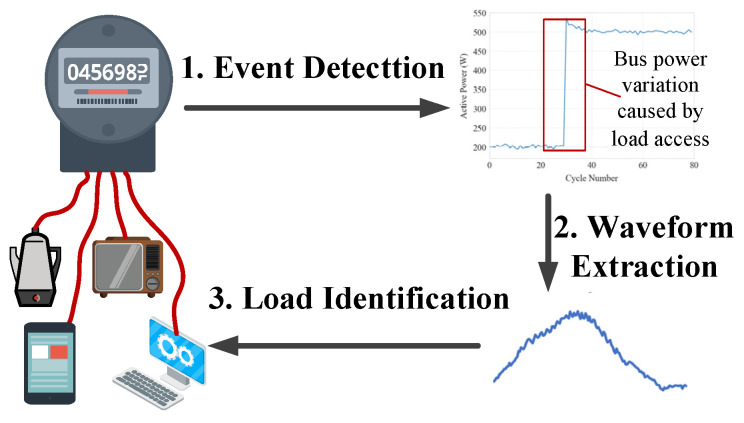
Workflow of an event-based, Non-Intrusive Load Monitoring approach.

The fundamental workflow of event-based NILM consists of three sequential steps: event detection, waveform extraction, and load identification, as depicted in Figure 1. The Event Detection stage identifies abrupt changes in the aggregated power curve, specifically during appliance activation or deactivation. The Generalized Likelihood Ratio Test (GLRT) [28] and the Cumulative Sum (CUSUM) method [29] are commonly implemented algorithms. These algorithms detect transient power variations by analyzing statistical deviations from baseline power consumption. When an event is detected, the corresponding current waveform I*_e_* is extracted from pre- and post-event segments based on Kirchhoff’s Current Law, the appliance-specific current is determined as follows:

(1) $$I_{e}=I_{a}-I_{b}$$

where *I_a_* represents the post-event current and *I_b_* indicates the pre-event current. This differential method isolates the target appliance’s electrical signature from the aggregated signal. Subsequently, the extracted waveform *I_e_* is processed through a pre-trained load identification model *f_Id_* (*I_e_*; *W*_0_), parameterized by *W*_0_, to classify the input into the corresponding appliance category *Y*_0_. The model parameters *W*_0_ are optimized during an offline training phase using a labeled dataset *D*_0_∼ {*I_e,0_*, *Y*_0_}, where gradient descent minimizes classification error over the training samples.

### 2.2. The Distributed Continuous Learning Setting in Practical NILM Systems

After the practical deployment of NILM systems, they typically encounter the following common scenarios: (1) Over time, clients may acquire new electrical appliances that were unseen during the training phase; (2) models trained on specific scenarios (e.g., residential settings) may require adaptation to different operational environments (e.g., industrial applications). The resulting shifts in data distribution across application scenarios can lead to performance degradation of the model. Consequently, NILM systems generally necessitate continuous knowledge updates to maintain optimal identification accuracy. Furthermore, in real-world implementations, apart from a limited amount of centrally labeled data, the newly available learning data is typically distributed across a subset of clients. These decentralized datasets can be formally represented as *D*_1_~{*I_e_*^1^, *Y*_1_},…, *D_k_*~{*I_e_*^k^, *Y_k_*}, where *k* represents the number of clients capable of local model updates.

Traditional sequential learning framework would utilize these datasets *D*_1_,…, *D_k_* sequentially to iteratively enhance a single global load identification model, updating its parameters from $W_{0}$ to *W_k_*. The proposed framework diverges fundamentally: each learnable client independently modifies a local copy of the initial model parameters *W*_0_ using their respective dataset (e.g., Client *i* updates *W*_0_ → *W_i_*), thus maintaining learned knowledge as distinct parameter sets *W* = [*W*_1_,…,*W_k_*]. This approach ensures that new data knowledge from learnable clients are preserved without catastrophic forgetting. The core challenge becomes the development of an adaptive model portfolio *W_i_*′ for each client (including those without local training capabilities) and enabling robust fusion-based identification across this portfolio.

## 3. Materials and Methods

### 3.1. Confidence-Based Collaborative Distributed Continual Learning Framework

**Figure 2:**
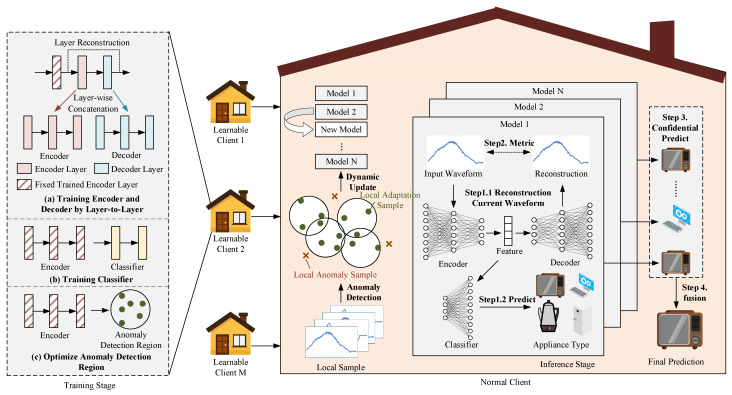
Confidence-based, collaborative, continual learning framework. **Left panel**: Training process of the proposed LWDS-AE model and anomaly detection region on each learnable client. Dynamically updated model portfolio based on client-side anomaly detection outcomes. **Right panel**: Reconstruction deviation-based confidence fusion identification process.

The proposed CBCDCL framework, depicted in Figure 2, comprises several key components. The initial phase involves local learning for clients with newly labeled data, incorporating four essential modules shown in the left panel: the encoder module, which converts raw input data into high-dimensional latent representations to enhance feature expressiveness; the decoder module, which reconstructs encoded features into input space for confidence assessment; the classification module, which generates appliance category predictions from encoded features; and the anomaly detection region, which identifies anomaly data in target clients to facilitate updates of client-side model portfolios. When new clients or data emerge, the client performs the described training process locally. Following local training completion, these module parameters are distributed to other clients within the NILM system. Recipient clients subsequently update their local model portfolios (central panel) by evaluating newly received models against a minimal-anomaly sample criterion—replacing existing models only when candidates demonstrate lower anomalies on local data. During appliance identification (right panel), all models within the client’s local model portfolio first generate decoded outputs (reconstructions) and classification outputs. The Euclidean distance between decoded outputs and raw inputs functions as a confidence metric, with classification results accepted only if this distance remains below a predefined threshold. When multiple valid outputs exist, multi-model voting determines the final prediction; if no outputs meet the threshold, all models contribute to the collective decision.

### 3.2. Construction of Layer-Wise Dual-Supervised Autoencoder Model

To effectively extract current waveform features of household electrical loads, this paper proposes an autoencoder model based on a layer-wise dual-supervised learning strategy (LWDS-AE). The encoder module and decoder module in LWDS-AE each contain three fully connected (FC) layers, while the classification module consists of two FC layers. The proposed layer-wise dual-supervised learning strategy optimizes the encoder and decoder module parameters through sequential training. As shown in the upper-left section of Figure 2, each encoder layer and its corresponding decoder layer undergo individual training. Upon completing one layer’s training, its parameters are fixed before advancing to the next layer until all layers achieve full optimization.

The loss function for each layer combines unsupervised reconstruction loss $L_{\mathrm{rcs}}$ and supervised classification loss $L_{\mathrm{clf}}$. The reconstruction loss calculates the Euclidean distance between the input $h_{i}$ and the reconstructed output $y_{i,\mathrm{rcs}}$ of the current layer, establishing hierarchical feature representations capable of accurate input restoration. Concurrently, the classification loss is determined by a two-layer FC classification module connected to the encoder output, which aligns the learned features with household load categories through task-specific supervision. This dual-supervised strategy maintains a balance between feature fidelity and discriminability, addressing the limitations of conventional unsupervised autoencoders that lack explicit category guidance. The loss function for each layer is defined as follows:

(2) $$Loss=\alpha L_{\mathrm{rcs}}+\beta L_{\mathrm{clf}}$$

(3) $$L_{\mathrm{rcs}}=\frac{1}{N_{s}}\sum_{i=1}^{N_{s}}{\left\|h_{i}-y_{i,\mathrm{rcs}}\right\|}^{2}$$

(4) $$L_{\mathrm{clf}}=-\frac{1}{N_{s}}\sum_{i=1}^{N_{s}}\sum_{c=1}^{N_{c}}y_{i,c}\log(p_{i,c})$$

where 

$y_{i,c}$ is the ground truth label of the input,

$p_{i,c}$ is the predicted probabilities of the classification module,

α,β is the loss weight coefficient, 

$N_{c}$ is the number of load categories,

$N_{s}$ is the number of training samples

After training the encoder-decoder model, to prevent exposure of sensitive information (e.g., user model parameters, weights, and architecture), the entire model is compiled into an executable file and distributed to other clients in the system to facilitate load identification tasks. Furthermore, a lightweight runtime protection mechanism is added to prevent reverse engineering of the model structure and logic. Specifically, we apply random scaling to the model prediction results y, which confuses the output probability distribution and reduces the risk of leaking the model’s decision boundaries, while preserving the original recognition accuracy of the model itself.

(5) $$y_{\mathrm{enc}}=y_{\mathrm{pred}}\cdot (1+\delta ),\;\delta \sim U(-0.05,0.05)$$

where $y_{\mathrm{pred}}$ denotes the original output of the model, representing unperturbed predictions. δ is a uniformly sampled random noise drawn from U(−0.05,0.05), which scales the output nonlinearly. $y_{\mathrm{enc}}$ indicates the encrypted output after perturbation.

### 3.3. Dynamically Update the Local Model Portfolio

As new data or clients emerge, each learnable client trains and outputs a new sub-model. For client devices receiving these models, limited computational performance and storage capacity make it impractical to store the growing volume of models transmitted from learnable clients. Thus, each client must strategically select and manage received models through an update method. This paper introduces an anomaly detection-based model portfolio management method to dynamically update the local model portfolio. Anomaly detection technology identifies samples that deviate from training data, thus preventing models from generating unreliable predictions. Based on this principle, the method evaluates which local data samples demonstrate anomalous characteristics relative to each candidate model. Subsequently, the system dynamically updates the local model portfolio by minimizing the occurrence of local anomaly samples under constrained model quantity conditions, thereby achieving optimal local model portfolios.

The implementation utilizes Support Vector Data Description (SVDD) [30] for model-specific anomaly detection. This technique establishes the minimal hypersphere containing training samples in a high-dimensional feature space, utilizing the sphere boundary as the decision criterion for anomalies. Its primary advantage lies in reducing data leakage risks by avoiding direct raw data transmission. Traditional SVDD typically applies to low-dimensional data, whereas high-frequency waveform data in power load monitoring generally exhibits high dimensionality. To address this challenge, this study proposes an encoder-SVDD integrated framework that optimizes anomaly detection boundaries based on encoded features from dimensionality-reduced representations. Specifically, the encoder module transforms high-dimensional input into compact latent features, enabling effective SVDD boundary optimization in the reduced feature space. This is mathematically represented as follows:

(6) $$\begin{array}{l} \underset{a,R,\xi }{\min}R^{2}+C_{p}\sum_{i}^{N_{s}}\xi_{i}\\ s.t.{\left\|c_{i}-a\right\|}^{2}\leq R^{2}+\xi_{i}\forall i,\xi_{i}\geq 0 \end{array}$$

where

$c_{i}$ is the load waveform sample;

a is the center of the hypersphere;

R is the radius of the hypersphere;

$C_{p}$ is the penalty coefficient;

$\xi_{i}$ is a slack variable.

Following the completion of encoder module training, each learnable client must optimize the hypersphere boundary using the encoded features of their local training data, as shown in the lower left panel of Figure 2. Through reformulation of this optimization problem into its Lagrangian dual form, the classic Sequential Minimal Optimization (SMO) [31] algorithm is employed to determine the center a and radius R:

(7) $$a=\sum_{i=1}^{N}\alpha_{i}c_{i}$$

(8) $$R=\sqrt{K_{v,v}-2K_{v,a}+K_{a,a}}$$

(9) $$K_{m,n}=K\left(c_{m},c_{n}\right)=e^{-\lambda {\left\|c_{m}-c_{n}\right\|}^{2}}$$

where $\alpha_{i}$ represents the Lagrange multiplier corresponding to sample $x_{i}$, most samples have $\alpha_{i}=0$, while a small subset of samples on the support region boundaries satisfies $0<\alpha_{i}<C_{p}$. *K* denotes the kernel function used to map input features into a high-dimensional space for enhanced optimization efficiency, where the subscript *v* indicates samples on the boundary.

After receiving anomaly detection parameters from learnable clients, each client computes the anomaly detection results $R_{\mathrm{new}}=[r_{1},r_{2},\ldots r_{N_{s}}],r\in \{-1,1\}$ for local data using Equations (10) and (11), where $N_{s}$ denotes the local data count.

(10) $$d_{i}=\sqrt{K_{i,i}-2K_{i,a}+K_{a,a}}$$

(11) $$r_{i}=\left\{\begin{array}{ll} 1 & \mathrm{if}d_{i}\leq R\\ -1 & \mathrm{if}d_{i}>R \end{array}\right.$$

A result where $r_{i}=-1$ indicates that a sample is anomalous. The model updating decision proceeds according to the following workflow: Let $R=[R_{1},R_{2},\ldots ,R_{N}]$ denote the anomaly detection outcomes of the current model portfolio containing *N* models.

Step 1: Calculate the baseline anomaly count $U_{o}$ for the existing portfolio using the indicator function *I*(⋅).

(12) $$U_{o}=\sum_{s=1}^{N}\prod_{R_{i}\in R}I(R_{i}(s)=-1)$$

Steps 2: Generate temporary model portfolios by creating all possible *N*−1-sized subsets of *R*, then integrate $R_{\mathrm{new}}$ into each subset to form $R_{\mathrm{temp}}^{k}$

(13) $$R_{\mathrm{temp}}^{k}=(R\backslash \{R_{k}\})\cup \{R_{\mathrm{new}}\}$$

Steps 3: Iteratively evaluate each $R_{\mathrm{temp}}^{k}$: calculate its anomaly count $U_{\mathrm{temp}}^{k}$, and replace the original portfolio if any $U_{\mathrm{temp}}^{k}$< $U_{o}$. If no improvement is observed, maintain the initial portfolio.

### 3.4. Multi-Model Confidence Identification Based on Reconstruction Deviation 

When an appliance activation event occurs, the previously described model portfolio facilitates collaborative identification. Given the variable performance and adaptability of individual models, simple voting mechanisms may result in situations where inferior models override superior ones. To address this challenge, this paper introduces a reconstruction deviation-based confidence identification methodology consisting of four steps (illustrated in Figure 2, right panel):

Step 1: Feature Encoding-Decoding: All models calculates classification prediction $y_{i}$ and reconstructed output $x_{i,\mathrm{rcs}}$ through the model’s encoder-decoder architecture:

(14) $$y_{i,\mathrm{pred}}=\mathrm{Classifier}(\mathrm{Encoder}(x_{i}))$$

(15) $$x_{i,\mathrm{rcs}}=\mathrm{Decoder}(\mathrm{Encoder}(x_{i}))$$

Step 2: Reconstruction Deviation Metric: Calculate the Euclidean distance between original input $x_{i}$ and the reconstructed output $x_{i,\mathrm{rcs}}$:

(16) $$d={\left\|x_{i}-x_{i,\mathrm{rcs}}\right\|}_{2}$$

Step 3: Confidence-Aware Model Screening: Designate models $M_{trust}$ as trustworthy when *d* falls below threshold *τ*, i.e., $M_{trust}=\{m_{k}|d_{k}<\tau \}$. This criterion ensures that only models exhibiting reconstruction consistency contribute to critical decisions.

Step 4: Adaptive Prediction Synthesis: Final predictions derive from hierarchical consensus - majority voting prioritizes outputs from $M_{trust}$ when non-empty, while unanimous voting across all models activates otherwise. This fallback mechanism ensures decision completeness while favoring credible predictions.

The methodology is based on a fundamental premise: Faithful waveform reconstruction (*d*→0) indicates precise feature alignment between test samples and the model’s training distribution. This alignment probabilistically correlates with reliable classification, as effective encoders capture invariant patterns essential for both reconstruction and discrimination tasks. By linking model credibility to reconstruction fidelity, the framework inherently weights predictions according to their feature-space coherence rather than mere output agreement.

### 3.5. Experimental Dataset and Evaluation Metrics

To comprehensively validate the proposed framework, this study utilizes two public datasets: PLAID [32] and WHITED [33]. The PLAID dataset, collected at a sampling frequency of 30 kHz across U.S. laboratories and 55 households, contains 1876 samples from 329 appliances spanning 16 categories. It remains the most widely utilized dataset for residential appliance identification. The WHITED dataset, acquired at 44.1 kHz across eight regions in Canada, Germany, Australia, and Indonesia, comprises 1258 samples covering 54 categories and 127 appliances. Notably, WHITED includes light industrial equipment such as electric drills, air compressors, and angle grinders, demonstrating significant inter-class variations among appliance categories.

To address the disparity in sampling frequencies (30 kHz vs. 44.1 kHz) and grid frequencies (60 Hz vs. 50 Hz) between the two datasets, the signals are uniformly down-sample to 150 sampling points per cycle using the SciPy library. This configuration corresponds to a 7.5 kHz sampling rate under 50 Hz grid conditions. Subsequently, event waveform sequences are extracted following the methodology outlined in Section 2 as input in the NILM model.

Model and framework performance is assessed through Accuracy (ACC) and Macro-averaged F1-score (F1-macro). Compared with ACC, F1-macro is more appropriate for classification tasks with imbalanced class distributions, as it equally weights contributions from each category. This metric becomes particularly critical in load identification scenarios where certain appliance categories (e.g., laptops and air conditioners) dominate sample counts, while others (e.g., blenders) are underrepresented. Let *N_s_* denote the total number of samples, *N_c_* the number of appliance categories, and *TP_c_*, *FP_c_*, *FN_c_* represent true positives, false positives, and false negatives for the *c*-th category, respectively. The computational formulas for *ACC* and F1-macro are defined as follows:

(17) $$ACC=\sum_{c=1}^{N_{c}}TP_{c}/N_{s}$$

(18) $$F_{macro}=\frac{1}{N_{c}}\sum_{c=1}^{N_{c}}\frac{2TP_{c}}{2TP_{c}+FP_{c}+FN_{c}}$$

## 4. Results

### 4.1. The Result of Model Performance Comparison

To evaluate the performance of the proposed LWDS-AE model, experiments were conducted on two datasets using 10-fold cross-validation. The datasets were divided into ten subsets, with one subset iteratively selected as the validation set and the remaining nine as the training set. The final performance metrics were averaged across all ten iterations. Five load identification models were compared: (1) 1DCNN [14]: A lightweight 1D convolutional neural network, balancing efficiency and identification accuracy. (2) AWRG [10]: A top-performing 2D convolutional neural network using amplitude-weighted recurrence graphs as inputs. (3) FCNN: A fully connected neural network mirroring the encoder and classifier structure of LWDS-AE. (4) Baseline DS-AE: The proposed architecture trained with conventional end-to-end strategies. For fairness, all models shared identical data splits, initialization methods, learning rates, and hyperparameters. The detailed structure of the proposed LWDS-AE model and learning parameter settings are in Appendix A.

As demonstrated in Table 1, the proposed LWDS-AE achieved superior accuracy and F1-macro scores across both datasets. In comparison to 1DCNN, LWDS-AE demonstrated markedly better identification metrics while requiring only a minimal parameter increase (0.2 Mb), making it suitable for practical deployment. The 2DCNN-based AWRG model achieved comparable performance to LWDS-AE on the PLAID dataset (matching accuracy) but demonstrated lower performance in other metrics and datasets. Importantly, AWRG required 76 times more computational resources and 20 times larger parameter sizes than LWDS-AE, restricting its practical implementation. The FCNN variant, despite sharing LWDS-AE’s forward architecture, produced the least favorable results, highlighting the essential role of training strategies and loss functions in model performance. Ablation studies confirmed the advantages of the proposed layer-wise training over end-to-end strategy, validating that the layer-wise dual-supervised strategy effectively extracts discriminative load features and develops robust load identification models.

### 4.2. Distributed Continual Learning Performance Under Identical Data Distribution

Section II formally defines the investigated scenario, in which real-time NILM systems are required to acquire new knowledge from incremental data distributed across partial clients. This scenario is further categorized into two sub-scenarios: new appliance learning under identical data distributions and new scenario learning under different data distributions.

For the new appliance learning scenario under identical data distributions, independent simulations are conducted on two datasets. Each dataset is divided into pre-training, incremental appliance, and test sets following a 1:2:7 ratio. The pre-training set comprises initially collectable appliance data typically limited in quantity, establishing preliminary model training and accelerating subsequent local learning processes. The incremental appliance set represents labeled training data generated during client-side operations, organized in groups of 20 appliances to represent learnable clients. The test set, similarly grouped by 20 appliances, represents ordinary clients with unseen appliances that cannot provide labeled training data. This configuration effectively replicates real-world appliance deployment and model application scenarios.

Several comparative methods are evaluated as follows: (1): Centralized learning (CL). This method collects data from learnable users to a central server for combined training with pre-training datasets. Constrained by privacy protection regulations, CL demonstrates low practical feasibility in real-world applications and thus serves only as a reference group to observe potential performance ceilings in this scenario. (2): Federated learning (FL), where all previously participating users engage in every subsequent incremental learning task. This approach incurs linearly increasing computational and communication costs as more users join, similarly exhibiting limited practical viability. (3): Sequential learning (SL), which transfers models sequentially between learnable users for localized adaptation. (4): SL + FL, combining method (3) with federated learning. This hybrid approach leverages localized collaboration between consecutive users to mitigate catastrophic forgetting while restricting collaboration scope to prevent the explosive resource consumption growth inherent in standard FL. (5): Elastic weight consolidation (EWC). This method records the Fisher information matrix of model parameters after each user’s learning phase as parameter importance metrics, subsequently constraining update magnitudes during continual learning. (6): Knowledge distillation (KD). After each learning task, teacher models are preserved and consistency losses are incorporated in subsequent tasks to maintain output alignment with these teachers. (7): Rehearsal (RH). This method retains partial historical samples (configured with a 500-sample memory capacity) for participation in new learning tasks.

**Figure 3:**
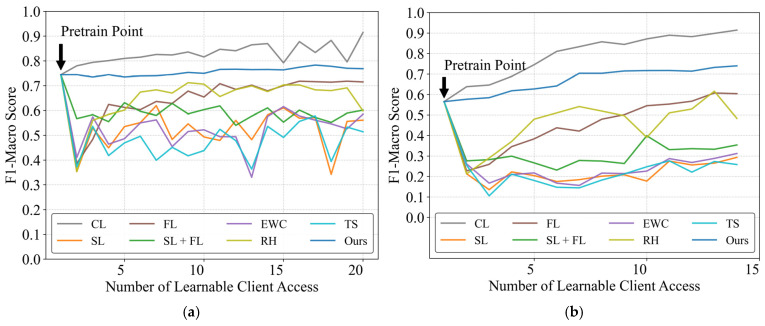
Performance evaluation of distributed continual learning methods under the same dataset. (**a**) PLAID dataset; (**b**) WHITED dataset.

Figure 3 illustrates the evolution of F1-macro scores on test clients in two datasets as learnable clients are progressively incorporated. Notably, only the framework demonstrates sustained performance improvement relative to the initial pre-training baseline, excluding CL. While unable to match CL’s performance, the proposed framework shows consistent upward trends, particularly evident in the WHITED dataset where initial performance improved from 0.565 to 0.739. This enhancement stems from WHITED’s greater appliance diversity and inter-appliance variability, where the proposed framework effectively leverages distributed model knowledge to broaden appliance coverage.

Other methods exhibited performance fluctuations and degradation across both datasets. Among these, sequential learning (SL) without constraints showed the most significant performance decline. The SL approach combined with federated learning demonstrated superior performance compared to standalone SL, as the federated learning process integrates information from consecutive users, thereby alleviating catastrophic forgetting. When comparing three fundamental continual learning methods (EWC, TS, and RH), both sample-preserving rehearsal and EWC enabled progressive knowledge accumulation with gradual performance improvements, though rehearsal displayed more pronounced performance volatility. The TS method underperformed, showing accuracy comparable to pure sequential learning, indicating that knowledge distillation strategies fail to achieve sustained multi-user knowledge accumulation. The proposed CBCDCL architecture circumvents parameter overwriting by maintaining separate model parameters and learned knowledge for each client, thereby eliminating performance degradation. While unrestricted FL outperforms SL, its linearly increasing communication and computational demands with growing learnable clients prove impractical for real-world sustainability.

This paper further analyzes and compares the real-world usability of the aforementioned methods, including computational costs during the training phase, communication transmission overhead, as well as storage and computational consumption during the inference phase. To facilitate comparative analysis, we assume all new clients contain one training sample and one test sample, with the number of training iterations set to one. The resource consumption characteristics during training and inference for each method are summarized in Table 2, where *N* represents the total number of learnable clients. The table shows that both centralized learning and federated learning exhibit quadratic growth in computational costs during the training phase as the number of clients increases. Regarding transmission resources, centralized learning requires transferring all raw data *D*, while federated learning necessitates *I* rounds of parameter exchange during each learning process, typically requiring at least dozens of exchanges to converge. In contrast, sequential learning and the proposed method demonstrate greater efficiency during the inference phase. Whenever a new learning task needs to be executed, only a single execution on learnable clients is required. Similarly for transmission, only the model parameters need to be transferred to other clients after training completion, significantly reducing parameter transmission costs compared with federated learning. From the inference perspective, centralized learning, federated learning, and sequential learning all maintain a single global model, meaning each client only needs to store and compute one model. However, the proposed method requires each client to store and compute *M* models, which represents a disadvantage compared to other approaches. Compared to the training process which often requires multiple iterations, each model in the proposed framework only performs a single forward pass during inference. Therefore, this inference overhead remains manageable. To substantiate this, we provide quantitative metrics for memory usage and latency per inference of our method during the inference phase to demonstrate feasibility for large-scale deployment. The primary memory concern here is storage memory, as during actual runtime, models can be loaded sequentially, requiring only one model’s runtime memory footprint at a time. The key variable affecting resource consumption is the model capacity limit (the maximum number of models). Thus, we tested the storage memory consumption and inference time for a single recognition task under different model capacity limits.

As shown in Table 3, higher model capacity limits incur greater memory overhead. Typically, relying solely on a microcontroller’s onboard memory (using STM32F407 as an example) is insufficient to meet the storage demand. Therefore, it necessitates an additional memory chip (e.g., W9825G6KSDRAM) to provide adequate storage. The execution times shown in the table were measured on a server equipped with an Intel(R) Xeon(R) Platinum 8160 CPU @ 2.10GHz. When deploying these methods on microcontroller-based smart meters or sockets, considering the substantial performance gap between such a server and a microcontroller, the inference computation time difference is estimated to be roughly three orders of magnitude. Consequently, inference time for our method on a microcontroller would be on the order of seconds. Despite this, as indicated by the model computational complexity comparison results in Table 1, the proposed method still achieves faster inference than the current state-of-the-art method, AWRG. This is primarily because the proposed models themselves are nearly two orders of magnitude smaller in terms of FLOPs. Naturally, due to significant differences in components between servers and microcontrollers, this estimated magnitude difference warrants further validation. We will subsequently deploy and test our method on the smart sockets we are developing for more concrete measurements.

### 4.3. Distributed Continual Learning Performance Across Scenarios

The previous experiment involved data partitioning within the same dataset, enabling the pre-trained model to establish robust baseline identification performance. However, real-world applications often involve operational scenarios where pre-collected appliances differ significantly from subsequently added or tested appliances, resulting in substantial distributional discrepancies. To simulate this scenario, two distinct datasets from different operational contexts are utilized: PLAID (residential electricity consumption) and WHITED (industrial and commercial electricity consumption). The experimental framework remains consistent with the prior setup, except that when dataset A is selected for pre-training, the incremental appliance and test sets are drawn from dataset B and partitioned in a 3:7 ratio.

**Figure 4:**
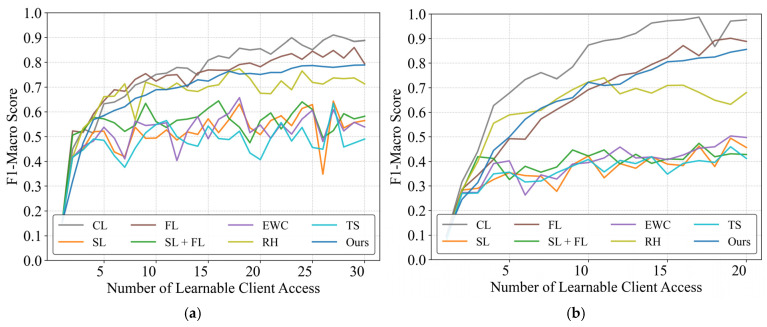
Performance evaluation of distributed continual learning methods across scenarios. (**a**) PLAID dataset (Pre-trained on WHITED); (**b**) WHITED dataset (Pre-trained on PLAID).

Figure 4 presents the cross-scenario experimental results. Under high distributional discrepancy conditions, the pre-trained model nearly fails entirely, exhibiting extremely low initial performance. Consequently, almost all methods achieve some improvement over the baseline. This scenario primarily evaluates the capacity of each method for continual knowledge acquisition and accumulation under significant distribution shifts. As shown in the figure, the framework demonstrates sustained performance enhancement as learnable client models are progressively incorporated, ultimately reaching 88.8% and 87.7% of CL’s performance levels in the respective scenarios.

For most sequential learning or basic continual learning methods, performance ceases to improve significantly after training on approximately five users, entering a phase of severe fluctuations that ultimately causes final accuracy to fall well below the minimum usability threshold. Only rehearsal demonstrated notable performance, achieving results comparable to our proposed method during the initial ten-user learning phase. However, beyond this point, rehearsal also entered a volatile performance regime due to its sample memory constraints, failing to achieve sustained knowledge accumulation. Interestingly, conventional federated learning (FL) marginally surpasses CBCDCL in this context. This superiority stems from FL’s requirement for previously trained clients to participate repeatedly in subsequent learning tasks, effectively providing additional training opportunities. However, this advantage incurs linearly increasing communication and computational resource demands as the number of learnable clients grows. Additionally, excessive learning tasks may disrupt clients’ primary monitoring functions. In contrast, CBCDCL achieves comparable performance with single-pass learning from clients, significantly reducing resource utilization.

### 4.4. Validation of Proposed Confidence Identification Strategy

In the proposed framework, as the trained model for learnable clients is preserved, the most essential component is the confidence assessment based on reconstruction deviation for enabling collaborative identification across multiple models. The effectiveness of the proposed strategy is validated by comparing the identification performance of different model aggregation strategies under identical model portfolios. The evaluated strategies include the following: (1) model output confidence assessment, where predictions are accepted only if their probability exceeds a predefined threshold [34]; (2) majority voting, where the final prediction is determined by the most frequent class label among all models; (3) model parameter averaging, which synthesizes a unified model by averaging parameters from all individual models for prediction.

**Figure 5:**
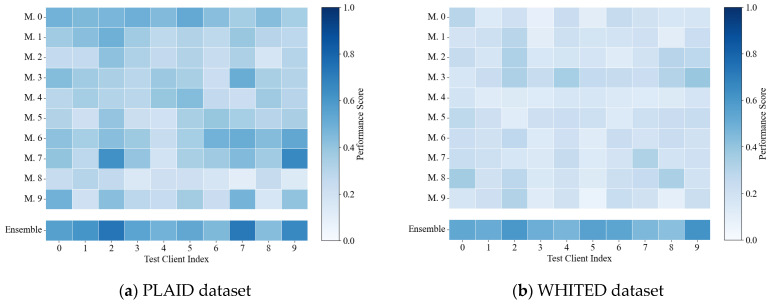
Standalone performance of individual models on test clients.

Table 4 presents the identification performance of these aggregation strategies under identical model portfolios. The proposed reconstruction deviation-based confidence identification strategy exhibits significant advantages over alternative strategies, validating its effectiveness. Additionally, upon analysis of individual models’ standalone performance on test clients, as illustrated in Figure 5, no single model achieves satisfactory identification performance across both datasets. However, the proposed confidence aggregation strategy based on reconstruction metrics consistently outperforms individual models, suggesting its effectiveness derives from integrating complementary knowledge across diverse models rather than relying on any particular model.

### 4.5. Validation of Proposed Model Portfolio Management Method 

**Figure 6:**
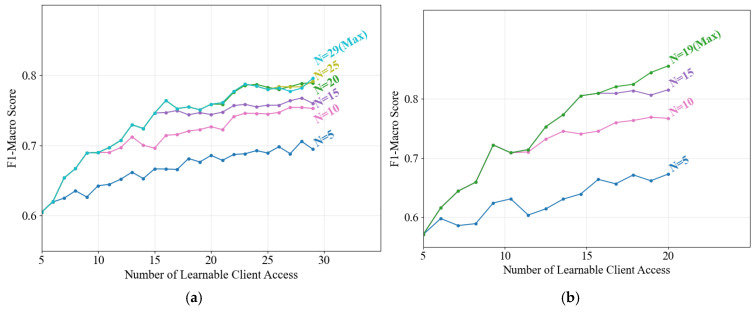
Performance evolution of updated model portfolios under varying model capacity limits via the proposed method. (**a**) PLAID dataset; (**b**) WHITED dataset.

Given the limited storage and computational resources of practical monitoring devices, this study proposes an anomaly detection-based model portfolio management method to dynamically update local model groups. Load identification performance is evaluated under varying model capacity limits (denoted as *N*, the maximum number of models retainable per client) across two cross dataset scenarios, as illustrated in Figure 6.

The identification performance shows consistent improvement longitudinally as learnable client models are incrementally incorporated, regardless of the preset *N*. This demonstrates the effectiveness of the proposed portfolio update strategy in selectively replacing existing models with superior alternatives to enhance local identification capabilities, thereby addressing NILM systems’ long-term operational requirements. Horizontally, higher model capacity limits correlate with improved final performance, albeit at the expense of increased storage and computational overhead. A balanced trade-off can be achieved by setting moderate *N* values—for instance, *N* = 20 for the PLAID dataset. Compared to retaining all models, this configuration reduces storage consumption by 31% while incurring only a 0.83% performance degradation, representing an acceptable compromise.

### 4.6. Practical Validation of the Proposed Framework

**Figure 7:**
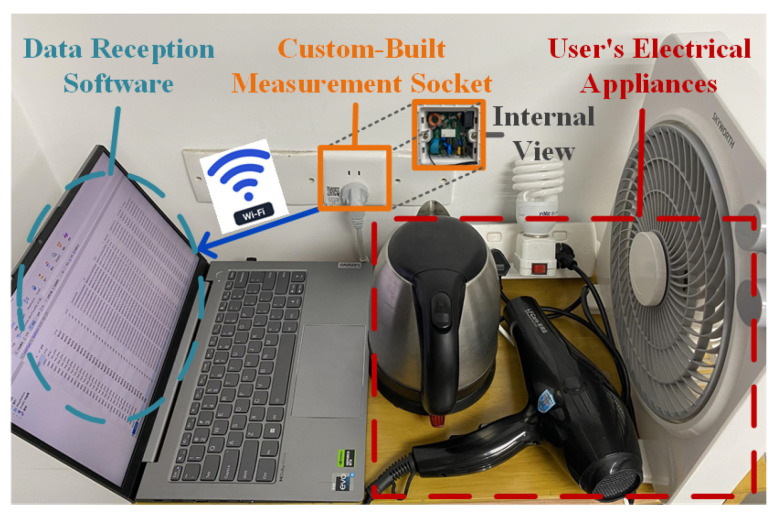
Practical implementation and evaluation using the measurement socket.

**Figure 8:**
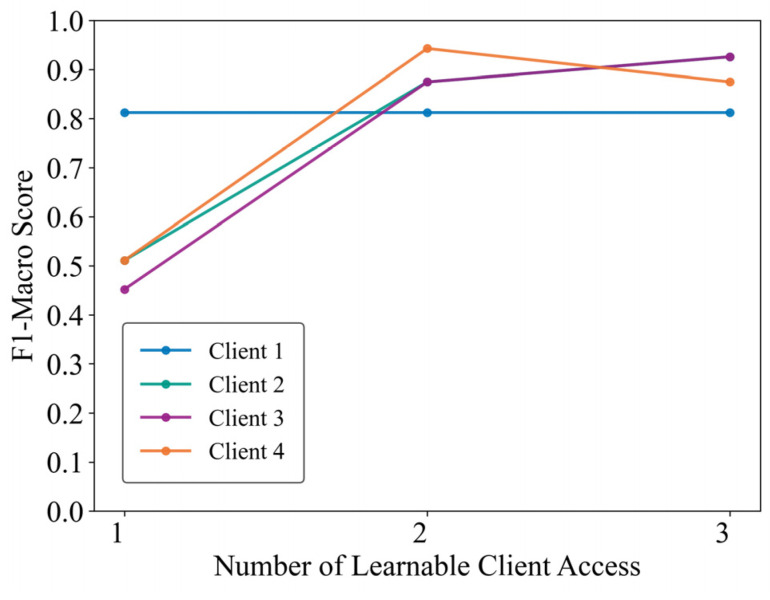
Performance evaluation of real-world clients.

To verify the practical applicability of the proposed framework, a high-frequency sampling measurement socket is developed and deployed, as illustrated in Figure 7. The outlet employs an STM32F407 microcontroller (STMicroelectronics, Geneva, Switzerland) as the edge computing core, utilizes a BL0956 chip (Belling, Shanghai, China) for high-frequency voltage and current acquisition, and integrates an ESP32 Wi-Fi module (Espressif Systems, Shanghai, China) for feedback transmission of identification results. A power strip facilitates connections to multiple appliances, simulating client bus data. A total of 33 physical appliances (e.g., battery chargers, fans, hair dryers, electric kettles, laptops, and lighting fixtures) are collected from four distinct locations. Appliances from one location served as test clients, while those from the remaining three locations functioned as learnable clients. Models trained independently on learnable clients were embedded into the outlets, followed by manual switching tests on the test clients’ devices. Experimental results, received via a computer-hosted upper monitor (Figure 8), demonstrate that clients 2, 3, and 4 achieved progressive identification performance improvements—reaching approximately 90%—as learnable client models were incrementally integrated. Client 1 attained high identification performance immediately after incorporating the first learnable client model. Subsequent models from the other two locations, however, provided limited additional gains due to significant data distribution discrepancies, resulting in stabilized performance. Nonetheless, the participation of learnable clients consistently enhanced overall identification performance within the proposed framework.

### 4.7. The Result of Hyperparameter Sensitivity Experiment

**Figure 9:**
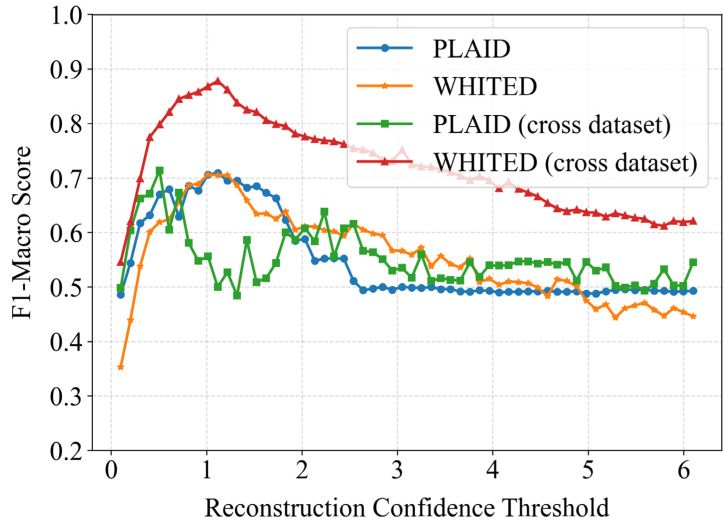
The impact of different confidence thresholds on load identification performance.

The confidence threshold based on reconstruction bias significantly impacts client-side load identification results. This study further investigated how different thresholds affect identification performance under both same-data-distribution and cross-dataset scenarios, maintaining experimental settings consistent with previous studies. As shown in Figure 9, all experimental groups exhibited a general trend of initial performance improvement followed by decline. This occurs because overly lenient/large thresholds may allow underperforming models to participate in identification, potentially introducing errors, while overly strict/small thresholds might exclude eligible models from participating in some samples. Therefore, appropriate threshold selection is crucial according to specific conditions.

Further analysis revealed that under same-data-distribution conditions, both PLAID and WHITED datasets achieved optimal performance near threshold = 1. In cross-dataset scenarios, WHITED as the target dataset similarly showed peak performance around threshold = 1, while PLAID as the target dataset achieved optimal results near threshold = 0.5. Notably, the PLAID dataset exhibited more pronounced performance volatility during threshold adjustment. This suggests greater threshold sensitivity in cross-dataset applications involving PLAID, potentially due to its richer appliance diversity compared to WHITED. The pre-trained models from WHITED might inadequately represent PLAID’s complex feature space, leading to differentiated optimal thresholds. For practical implementation, we recommend obtaining optimal threshold settings through limited batch sample testing. Specifically, after users update their local model combinations, they should utilize a pre-annotated calibration dataset for testing to determine the optimal threshold for the current model ensemble.

### 4.8. Targeted Ablation Experiments

To isolate the contribution of each proposed module, we conducted targeted ablation experiments. The experimental settings remained consistent with the earlier configuration, including identical data partitioning and hyperparameters. The maximum number of learnable user models was capped at 20 by default, with the final identification performance after incorporating all learnable users as the evaluation metric. For each ablation, only one specific module was modified:

(1) Removal of the layer-wise dual-supervised auto-encoder (LWDSAE): Replaced with conventional end-to-end auto-encoder learning.
(2) Removal of confidence-based fusion: substituted with a simple majority voting ensemble across all models.
(3) Removal of model quantity limitation: allowed clients to retain all received models without restrictions (disabling the anomaly detection-based model replacement mechanism).

Experiments were performed on both single-dataset and cross-dataset scenarios. Results are presented in Table 5 below.

In all four experiments, replacing the proposed LWDSAE strategy with conventional end-to-end auto-encoding consistently underperformed, despite sharing identical model inference architectures. This demonstrates that LWDSAE effectively extracts discriminative features essential for both load identification and confidence estimation tasks. Similarly, replacing confidence-based fusion with majority voting caused significant performance degradation. This occurs because models trained on disparate user data distributions exhibit poor cross-user generalizability. Our confidence fusion mechanism mitigates this issue by filtering model predictions based on reliability, reducing interference from erroneous outputs. Removing the model quantity limitation via anomaly detection had a marginal impact on performance. The confidence estimation stage inherently limits the influence of additional models on prediction quality; the primary impact manifests in increased computational and storage overhead. Notably, this ablation had no effect on the WHITED dataset due to its low number of learnable users (<20), preventing the model replacement strategy from activating. The relationship between model quantity limits and identification performance is further illustrated in Figure 6.

## 5. Discussion

To address distributed continual learning challenges in practical NILM scenarios, this paper proposes a confidence-based, multi-model, collaborative distributed continual learning framework. Under this framework, learnable clients conduct local learning while other system clients can acquire newly updated models to construct optimal local model portfolios, thereby enhancing local identification performance. To support load identification tasks, we designed a layer-wise, dual-supervised autoencoder model. Compared with current state-of-the-art load identification models, the LWDSAE model achieves superior performance across four metrics on two benchmark datasets. Notably, the model demonstrates lower complexity than AWRG and 1DCNN architectures, with parameter size merely 0.2 Mb higher than 1 DCNN while significantly outperforming it in accuracy. This indicates the proposed model satisfies both performance requirements and lightweight deployment needs.

For distributed incremental learning tasks, conventional continual learning and federated learning methods using single-model structures often suffer from catastrophic forgetting. To resolve this, we propose a confidence-collaborative distributed continual learning method employing multi-model structures to preserve knowledge from diverse clients. Through reconstruction bias evaluation of each model’s prediction reliability, the proposed method achieves confidence-weighted fusion identification across model portfolios. Cross-distribution experiments demonstrate performance reaching 88% of centralized learning benchmarks, effectively integrating new client knowledge while preserving privacy.

The primary advantage of the proposed method lies in its low training overhead, requiring only single-pass learning per learnable client. The main limitation resides in higher computational resource demands during inference, as each client must store multiple external models for local inference and load identification. To address this, we developed an anomaly detection-based dynamic model portfolio management method that evaluates new model compatibility with clients, enabling automated model replacement decisions for knowledge updating and cumulative learning.

In summary, this paper’s proposed framework demonstrates stable continual performance improvement capabilities in distributed learning scenarios, significantly outperforming existing learning approaches with strong practical value. Furthermore, we validated the proposed method on two public datasets and through deployment on an STM32 F407 microcontroller-based smart socket prototype, confirming its capability to meet practical NILM application requirements.

## 6. Conclusions

This study proposes a distributed continual learning framework for Non-Intrusive Load Monitoring, addressing challenges in knowledge updating and inter-client collaboration for real-world deployment. The main contributions are summarized as follows:

(1) A dual-layer, supervised, learning-based autoencoder model is developed for load identification, which achieves higher lightweight tatus, thereby advancing the practical deployment of NILM systems.
(2) A reconstruction deviation-based model confidence identification strategy is introduced to enable multi-model collaboration across clients, achieving the best-known performance in cross-model aggregation.
(3) A dynamic model composition update method is proposed to continually enhance local load monitoring performance under storage and computational constraints.

These innovations collectively provide a scalable solution for continuous performance enhancement in distributed NILM systems. Future work will focus on integrating knowledge distillation for model compression and extending the framework to industrial equipment monitoring scenarios.

## Figures and Tables

**Table 1:** Comparison of performance and complexity of various models.

Model	Performance				Complexity	
	PLAID		WHITED		Params (Mb)	FLOPs
	ACC	F1-Macro	ACC	F1-Macro		
1DCNN [14]	0.936	0.913	0.898	0.911	**0.85**	77,248
AWRG [10]	**0.974**	0.937	0.969	0.946	21.22	2,582,160
FCNN	0.916	0.868	0.850	0.856	1.05	**34,048**
DS-AE	0.917	0.838	0.859	0.869	1.05	**34,048**
LSDS-AE	**0.974**	**0.966**	**0.976**	**0.965**		

**Bold**: optimal in vertical comparison.

**Table 2:** Comparison of the real-world usability of the aforementioned methods.

	Training Phase		Inference Phase in Each Client	
	Calculations	Transmissions	Storages	Calculations
CL	$N^{2}$	N+D	1	1
FL	$N^{2}$	N×(N+1)×I	1	1
SL	N	N×(N−1)	1	1
Ours	N	N×(N−1)	*M*	*M*

**Table 3:** Memory usage and inference time under various model capacities.

Model Capacity	Memory Usage (Mb)	Inference Time (s)
5	5.25	0.004
10	10.50	0.008
15	15.75	0.011
20	21.00	0.014

**Table 4:** Identification performance comparison under different multi-model aggregation strategies.

Aggregation Strategy	Single Dataset				Cross Dataset			
	PLAID		WHITED		PLAID		WHITED	
	ACC	F1	ACC	F1	ACC	F1	ACC	F1
Output confidence	0.700	0.605	0.520	0.453	0.715	0.619	0.696	0.617
Majority voting	0.691	0.593	0.505	0.418	0.721	0.631	0.622	0.528
Parameter averaging	0.566	0.446	0.292	0.181	0.344	0.281	0.166	0.063
The proposed	**0.812**	**0.767**	**0.773**	**0.751**	**0.833**	**0.794**	**0.876**	**0.854**

**Bold**: optimal in vertical comparison.

**Table 5:** Targeted ablation experiment results.

Comparison	PLAID	WHITED	PLAID (c.d.)	WHITED (c.d.)
Proposed	0.812/0.767	0.773/0.750	0.833/0.794	0.876/0.854
Proposed *w*/*o* LWDS	0.724/0.660	0.551/0.488	0.725/0.679	0.678/0.607
Proposed *w*/*o* confidence fusion	0.691/0.593	0.506/0.418	0.720/0.631	0.622/0.528
Proposed *w*/*o* model portfolio limit	0.807/0.763	0.773/0.750	0.821/0.800	0.876/0.854

## Data Availability

The data presented in this study are available on request from the corresponding author. The data are not publicly available due to privacy.

## Appendix A

### Appendix A.1

**Table A1:** The detailed structure of the proposed LWDS-AE model.

Module	Structure
Encoder	FC: (150, 128) + ReLU
	FC: (128, 64) + ReLU
	FC: (64, 64) + ReLU
Decoder	FC: (64, 64)
	FC: (64, 128)
	FC: (128, 150)
Classifier	FC: (64, 32) + ReLU
	FC: (32, *N_C_*)

**Table A2:** Core and supplementary parameters in this paper.

Parameter Description	Value
Learning batch size	128
Learning rate	0.001
Pre-training epoch	100
Training epoch in learnable client	100
Optimizer	Adam
α in (2)	[1, 1, 0.5] for each layer
β in (2)	[0.5, 1, 1] for each layer
$\mathrm{Penalty}\;\mathrm{coefficient}\;C_{p}$ in (5)	1
λ in (8)	3
Confidential threshold *τ*	1

## References

[1] Hart G.W. (1992). Nonintrusive Appliance Load Monitoring. Proc. IEEE.

[2] Liu Y., Wang Y., Ma J. (2024). Non-Intrusive Load Monitoring in Smart Grids: A Comprehensive Review. arXiv.

[3] Schirmer P.A., Mporas I. (2023). Non-Intrusive Load Monitoring: A Review. IEEE Trans. Smart Grid.

[4] Dash S., Sahoo N.C. (2022). Electric Energy Disaggregation via Non-Intrusive Load Monitoring: A State-of-the-Art Systematic Review. Electr. Power Syst. Res..

[5] Yan L., Tian W., Han J., Li Z. (2022). Event-Driven Two-Stage Solution to Non-Intrusive Load Monitoring. Appl. Energy.

[6] Zheng Z., Chen H., Luo X. (2018). A Supervised Event-Based Non-Intrusive Load Monitoring for Non-Linear Appliances. Sustainability.

[7] Ji T., Chen J., Zhang L., Lai H., Wang J., Wu Q. (2025). Low Frequency Residential Load Monitoring via Feature Fusion and Deep Learning. Electr. Power Syst. Res..

[8] Elahe M.F., Jin M., Zeng P. (2022). Knowledge-Based Systematic Feature Extraction for Identifying Households with Plug-in Electric Vehicles. IEEE Trans. Smart Grid.

[9] Du L., He D., Harley R.G., Habetler T.G. (2016). Electric Load Classification by Binary Voltage–Current Trajectory Mapping. IEEE Trans. Smart Grid.

[10] Faustine A., Pereira L., Klemenjak C. (2021). Adaptive Weighted Recurrence Graphs for Appliance Recognition in Non-Intrusive Load Monitoring. IEEE Trans. Smart Grid.

[11] Mari S., Bucci G., Ciancetta F., Fiorucci E., Fioravanti A. (2022). A Review of Non-Intrusive Load Monitoring Applications in Industrial and Residential Contexts. Energies.

[12] Liu Y., Wang X., You W. (2019). Non-Intrusive Load Monitoring by Voltage–Current Trajectory Enabled Transfer Learning. IEEE Trans. Smart Grid.

[13] D’Incecco M., Squartini S., Zhong M. (2020). Transfer Learning for Non-Intrusive Load Monitoring. IEEE Trans. Smart Grid.

[14] Luo Q., Yu T., Lan C., Huang Y., Wang Z., Pan Z. (2023). A Generalizable Method for Practical Non-Intrusive Load Monitoring via Metric-Based Meta-Learning. IEEE Trans. Smart Grid.

[15] Wang L., Mao S., Wilamowski B.M., Nelms R.M. (2022). Pre-Trained Models for Non-Intrusive Appliance Load Monitoring. IEEE Trans. Green Commun. Netw..

[16] Zhou Z., Xiang Y., Xu H., Yi Z., Shi D., Wang Z. (2021). A Novel Transfer Learning-Based Intelligent Nonintrusive Load-Monitoring With Limited Measurements. IEEE Trans. Instrum. Meas..

[17] Lin J., Ma J., Zhu J., Liang H. (2022). Deep Domain Adaptation for Non-Intrusive Load Monitoring Based on a Knowledge Transfer Learning Network. IEEE Trans. Smart Grid.

[18] Dai S., Meng F., Wang Q., Chen X. (2024). DP2-NILM: A Distributed and Privacy-Preserving Framework for Non-Intrusive Load Monitoring. Renew. Sustain. Energy Rev..

[19] Lin J., Ma J., Zhu J. (2022). Privacy-Preserving Household Characteristic Identification With Federated Learning Method. IEEE Trans. Smart Grid.

[20] Ruder S. (2017). An Overview of Multi-Task Learning in Deep Neural Networks 2017. arXiv.

[21] McCloskey M., Cohen N.J., Bower G.H. (1989). Catastrophic Interference in Connectionist Networks: The Sequential Learning Problem. Psychology of Learning and Motivation.

[22] Kirkpatrick J., Pascanu R., Rabinowitz N., Veness J., Desjardins G., Rusu A.A., Milan K., Quan J., Ramalho T., Grabska-Barwinska A. (2017). Overcoming Catastrophic Forgetting in Neural Networks. Proc. Natl. Acad. Sci. USA.

[23] Aljundi R., Babiloni F., Elhoseiny M., Rohrbach M., Tuytelaars T. (2018). Memory Aware Synapses: Learning What (Not) to Forget. Proceedings of the European Conference on Computer Vision (ECCV).

[24] Li S., Su T., Zhang X.-Y., Wang Z. (2024). Continual Learning with Knowledge Distillation: A Survey. IEEE Trans. Neural Netw. Learn. Syst..

[25] Qiu L., Yu T., Lan C. (2024). A Semi-Supervised Load Identification Method with Class Incremental Learning. Eng. Appl. Artif. Intell..

[26] Shoham N., Avidor T., Keren A., Israel N., Benditkis D., Mor-Yosef L., Zeitak I. (2019). Overcoming Forgetting in Federated Learning on Non-IID Data. arXiv.

[27] Zhang C., Xie Y., Bai H., Yu B., Li W., Gao Y. (2021). A Survey on Federated Learning. Knowl.-Based Syst..

[28] Anderson K.D., Bergés M.E., Ocneanu A., Benitez D., Moura J.M.F. Event Detection for Non Intrusive Load Monitoring. Proceedings of the IECON 2012—38th Annual Conference on IEEE Industrial Electronics Society.

[29] Fang K., Huang Y., Huang Q., Yang S., Li Z., Cheng H. An Event Detection Approach Based on Improved CUSUM Algorithm and Kalman Filter. Proceedings of the 2020 IEEE 4th Conference on Energy Internet and Energy System Integration (EI2).

[30] Tax D.M.J., Duin R.P.W. (1999). Support Vector Domain Description. Pattern Recognit. Lett..

[31] Torres-Barrán A., Alaíz C.M., Dorronsoro J.R. (2021). Faster SVM Training via Conjugate SMO. Pattern Recognit..

[32] Medico R., De Baets L., Gao J., Giri S., Kara E., Dhaene T., Develder C., Berges M., Deschrijver D. (2020). A Voltage and Current Measurement Dataset for Plug Load Appliance Identification in Households. Sci. Data.

[33] Kahl M., Haq A., Kriechbaumer T., Jacobsen H.-A. WHITED—A Worldwide Household and Industry Transient Energy Data Set. Proceedings of the 3rd International Workshop on Non-Intrusive Load Monitoring.

[34] Moon J., Kim J., Shin Y., Hwang S. Confidence-Aware Learning for Deep Neural Networks. Proceedings of the International Conference on Machine Learning.

